# Supplementary material for: Genome structural variation in Escherichia coli O157:H7
Source: Microb Genom. 2021 Nov 9;7(11):000682. doi: 10.1099/mgen.0.000682 (PMC8743559; doi:10.1099/mgen.0.000682)
Supplement: Supplementary material 1 [file mgen-7-0682-s001.pdf]

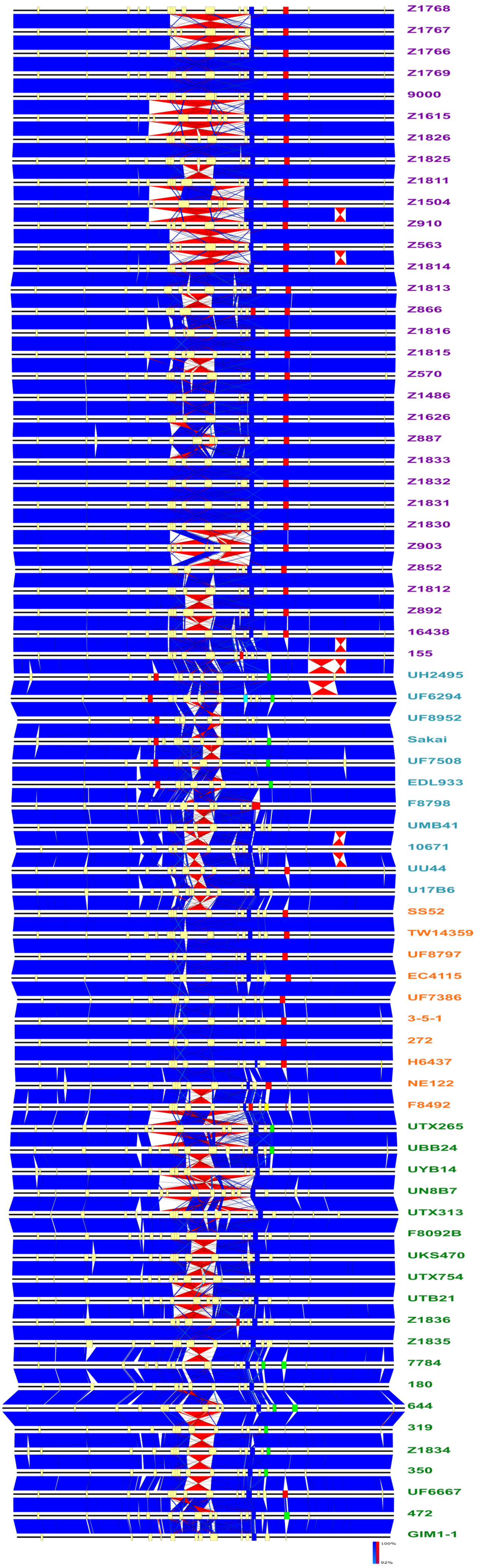

Figure S1. Pairwise whole genome comparisons of 72 *E. coli* O157 strains. The strains analysed cover the three major lineages: Lineage I (Light blue with sub-lineage Ic purple), Lineage I/II (orange) and Lineage II (green). Whole genomes (black lines) are centred by the replication terminus (Ter) and the loci of prophage (yellow boxes) and Stx prophage (ΦStx2c;blue ΦStx2a;red and ΦStx1;green) are shown. Direct (blue) and inverted (red) homology at a blast cut-off of 10,000 bp between strains are plotted.

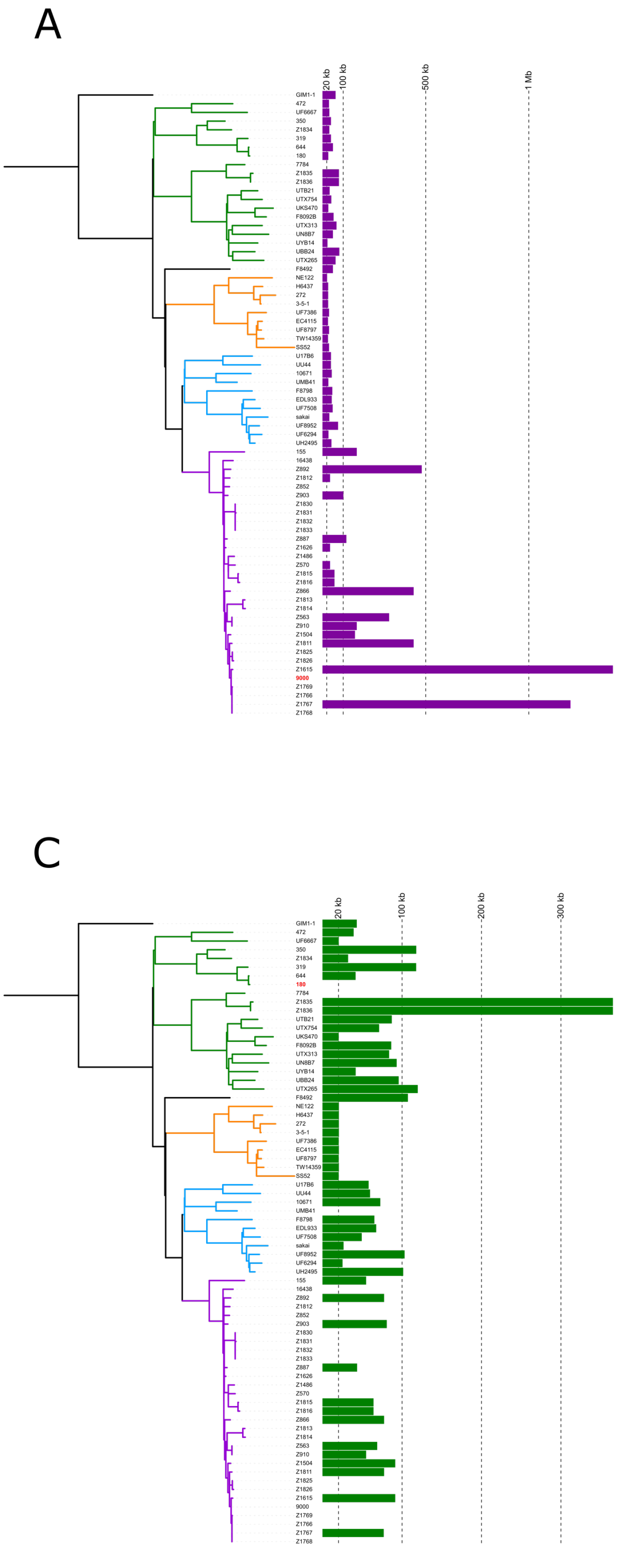

Figure S2. The mean size of chromosomal rearrangements ≥20 kb in *E. coli* O157 strains.

The phylogenetic distribution based on core gene relatedness of the 72 strains analysed from Lineage 1c (purple), Lineage 1a (blue) Lineage I/II (orange) and Lineage II (green) is shown. Bars at the tree tips show the mean size of LCRs ≥20 kb detected for strains relative to each reference (red bold). Reference strains used were: (**A**) 9000, Lineage 1c; (**B**) Sakai, Lineage Ia; (**C**) TW14359, Lineage I/II; (**D**) 180, Lineage II.

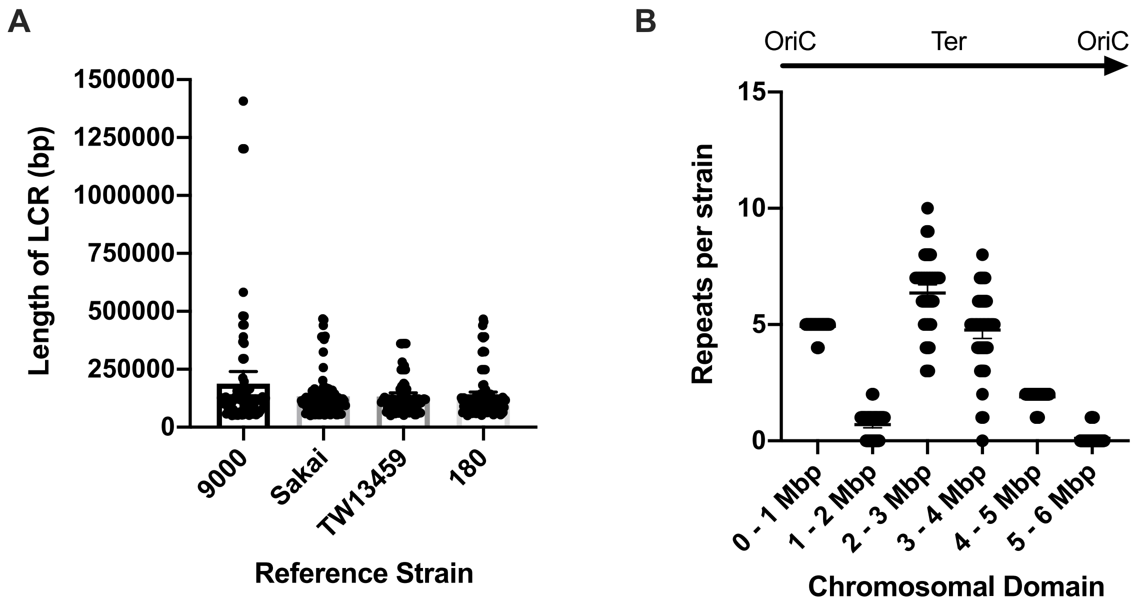

Figure S3. Length of detected LCRs and Ter bias of repeat sequences.

(A) Dot plot showing the length (bp) of all detected LCRs relative to four reference strains: 9000, Lineage 1c; Sakai, Lineage Ia; TW14359, Lineage I/II; 180, Lineage II. The mean length (bars) +/- 95 % CI are also shown. (B) Chromosomes were divided into 1 Mbp regions and the total number of repeats sequences detected in each strain within each 1 Mbp were plotted. The mean number of repeat sequences +/- 95 % CI are also shown.

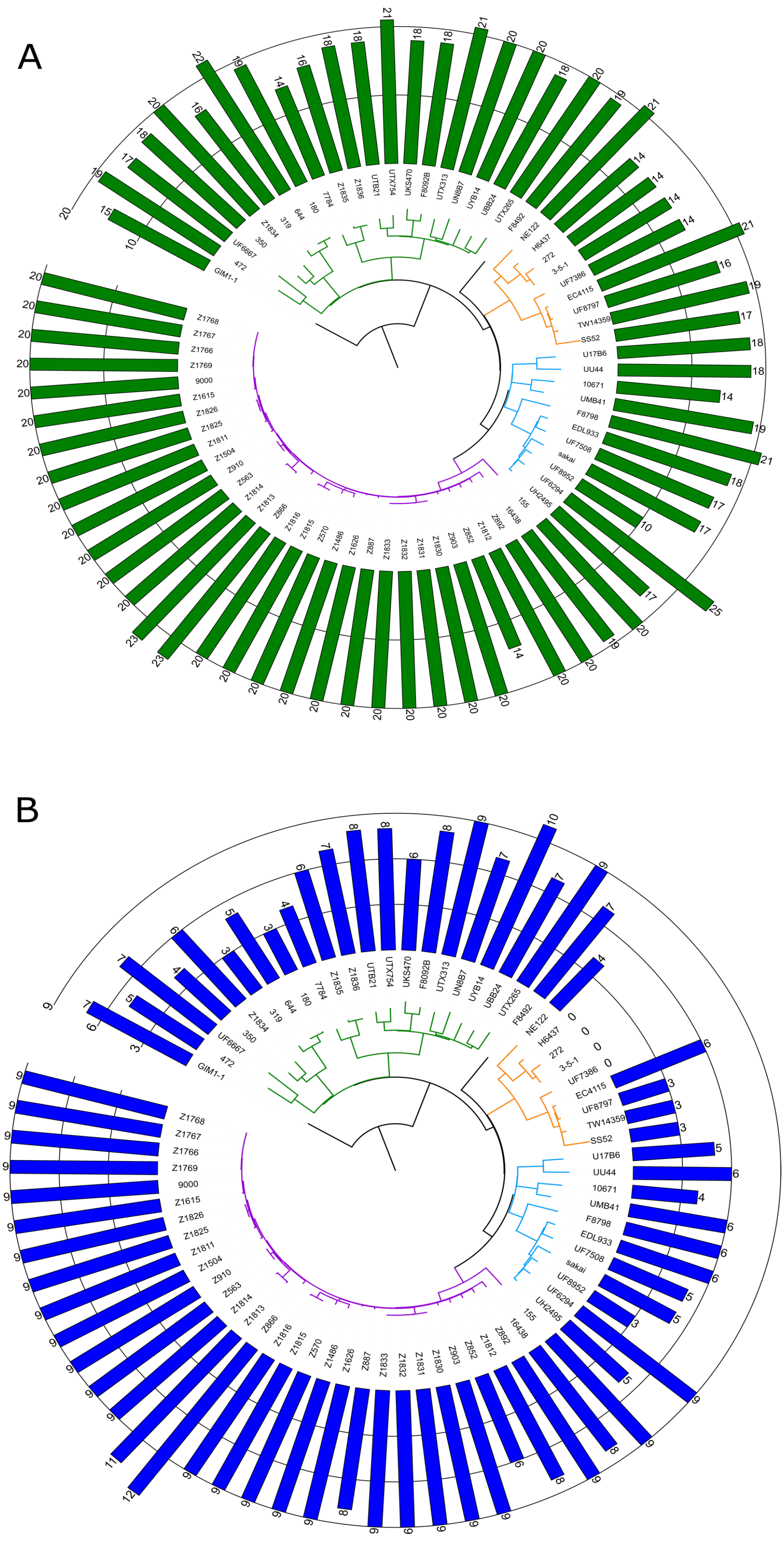

Figure S4. Total number of homologous repeat regions in 72 *E. coli* O157 strains.

The phylogenetic distribution based on core gene relatedness of strains from Lineage 1c (purple), Lineage 1a (blue) Lineage I/II (orange) and Lineage II (green) is shown. Bars at the tree tips show the total number of repeat homologous sequences ≥ 5 kb (**A**) and ≥ 8kb (**B**) detected in each strain.

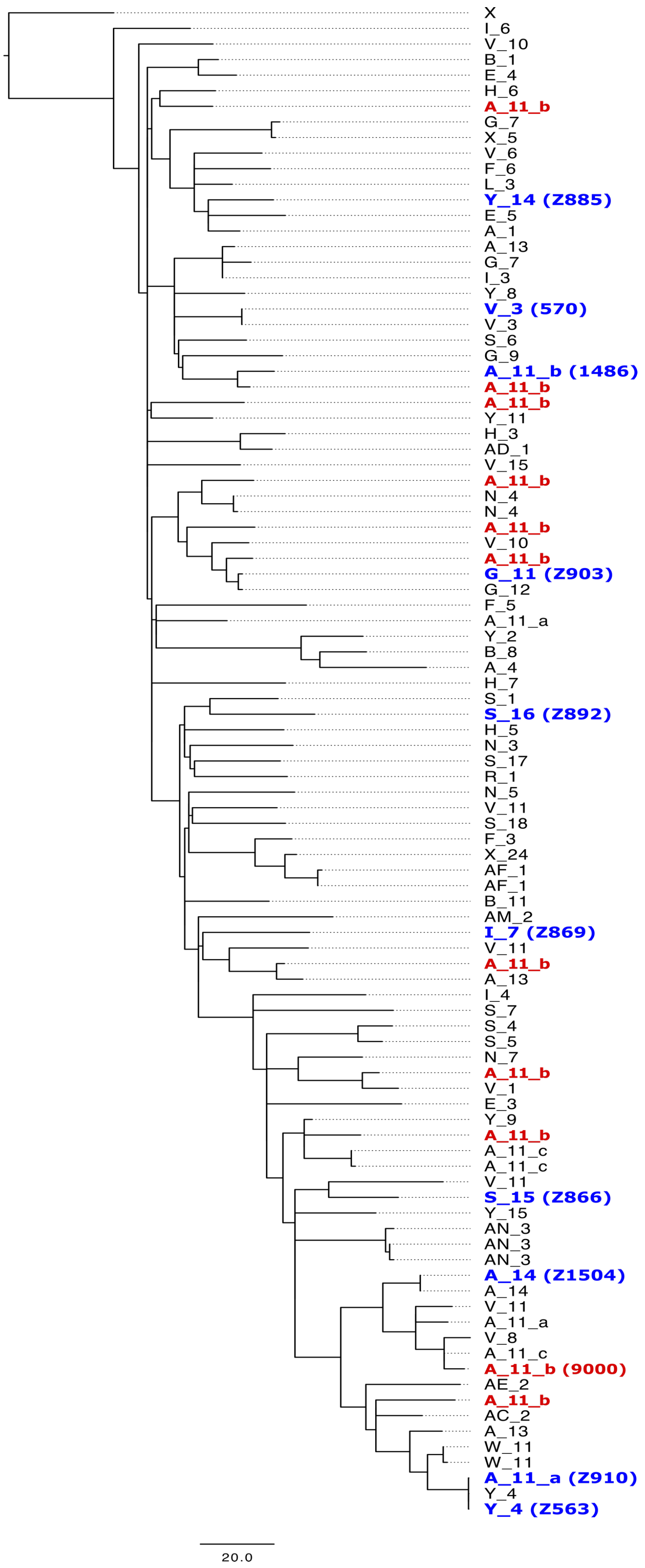

Figure S5. Phylogenetic distribution of PT21/28 strains in the UK. A core SNP based phylogeny of representative human and bovine PT21/28 strains from the UK is shown. PFGE profile types assigned by Scottish *E. coli* Reference Laboratory (SERL) are at branch tips for each strain. Strains selected for PacBio sequencing (blue) and those with PFGE profile C (A_11b) that occur throughout the cluster (red) are indicated. Scale bar indicates basepair substitutions.

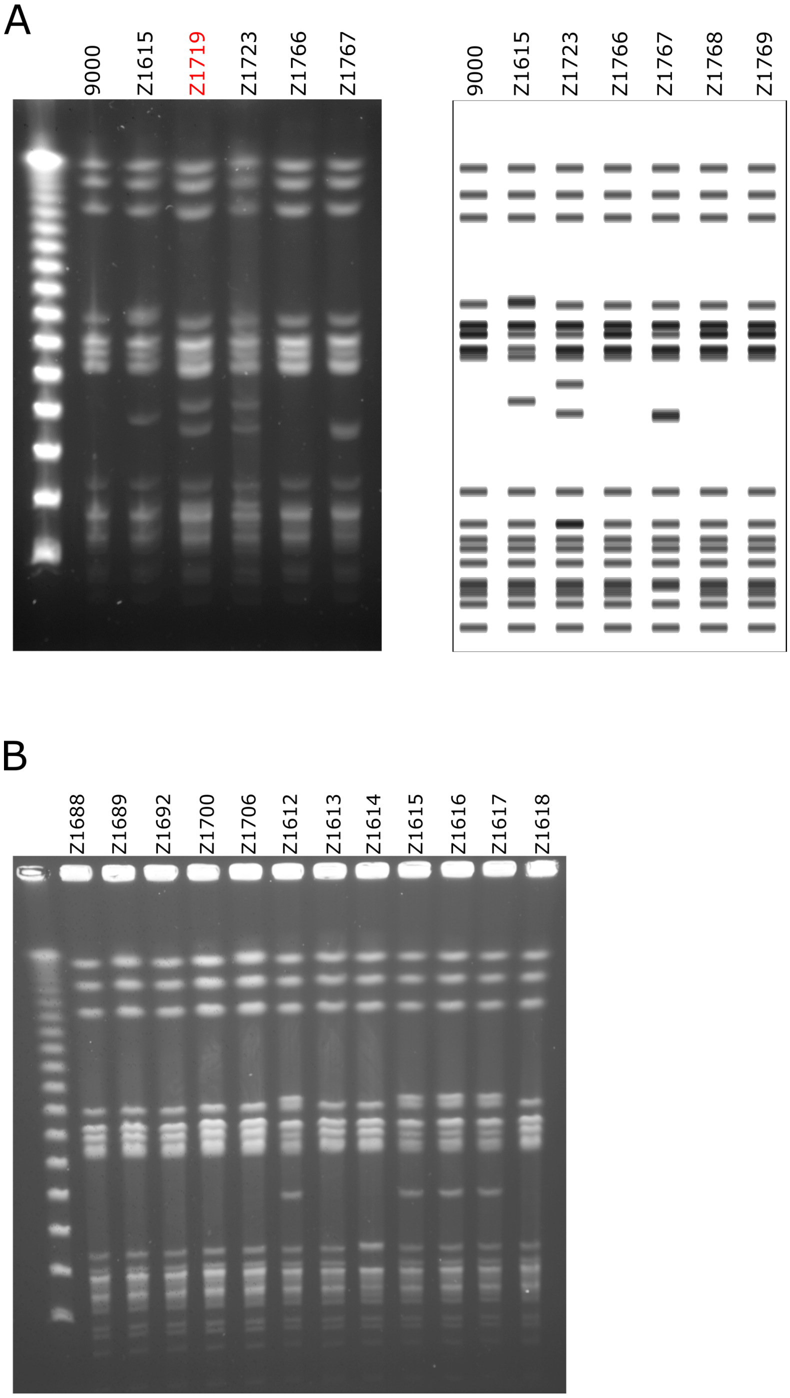

Figure S6. PFGE profiles of *E. coli* O157 PT21/28 strain 9000 derivatives recovered from cattle colonisation trials. (**A**) Comparison of PFGE and *in silico* generated AvrII restriction profiles of strains from Trial 1 (9000 and Z1615) and Trial 2 (Z1723, Z1766 and Z1767). A duplicate freezer stock of strain Z1723 (Z1719) was also analysed by PFGE. (**B**) PFGE profiles following AvrII digestion of Z1615 and 11 additional isolates recovered from trials where strain 9000 was used as an inoculum.

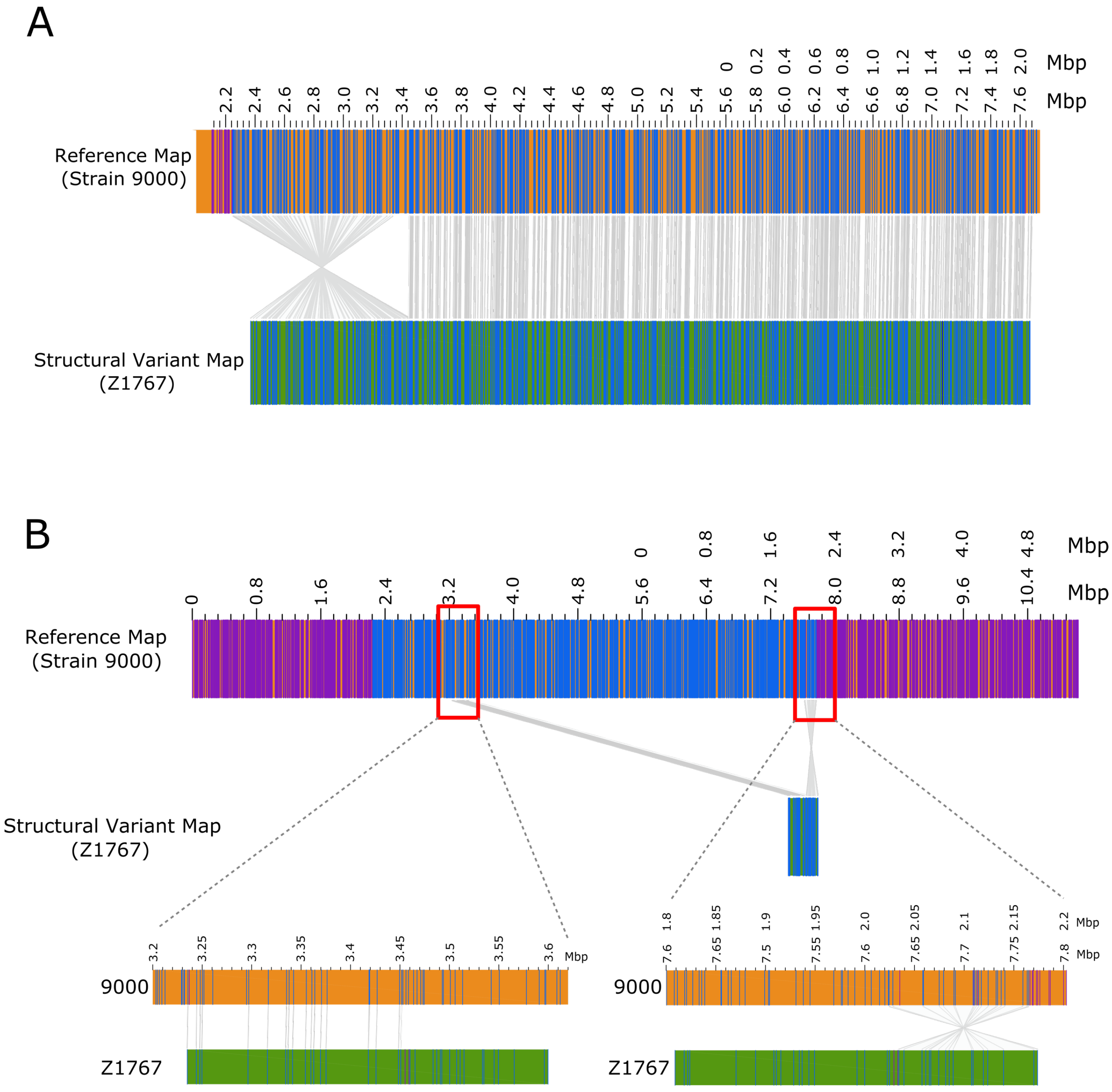

Figure S7. Optical mapping of *E. coli* O157 PT21/28 strain 9000 variant Z1767.

Structural variant (SV) analysis identified a 1.2 Mb inversion (**A**) and 145 kb inverted duplication (**B**) in the population of Z1767 relative to reference strain 9000. The genome map (orange) of reference strain 9000 and each Z1767 structural variant (green) are shown. Paired restriction sites (blue lines) are aligned between the reference and variant maps (grey lines). Unpaired restrictions (purple lines) outside aligned regions are also shown. The hybrid SV map containing the 145 Kb duplication aligned to two distinct regions of reference strain 9000 genome: 5' – 3' within ΦStx2c at 3.4 Mb and 3' – 5' at 1.95 Mb.

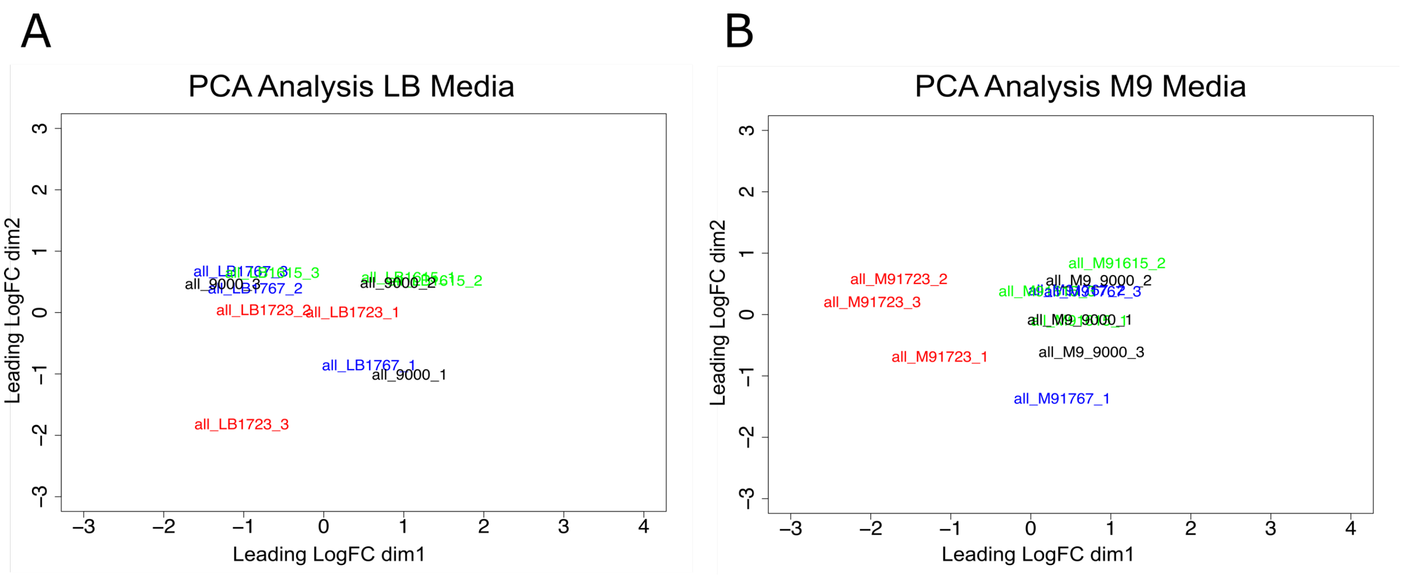

Figure S8. PCA analysis of RNAseq data generated for *E. coli* O157 PT21/28 strain 9000 derivatives. Expression (Log fold change) was compared for strains 9000 (black), Z1615 (green), Z1723 (red) and Z1767 (blue) grown in LB (A) and M9 (B) media. Three replicates of each strain were used for analysis.
